# Prospective Association of Symptoms of Depression and Anxiety with Pornography Viewing Frequency Among Young Adults

**DOI:** 10.1007/s10508-024-03024-y

**Published:** 2024-11-01

**Authors:** Chithra Singareddy, Sambid Shrestha, Amy Zheng, Bernard L. Harlow, Jessica L. Barrington-Trimis, Alyssa F. Harlow

**Affiliations:** 1 Department of Epidemiology, Boston University School of Public Health, Boston, MA USA; 2 Department of Population and Public Health Sciences, University of Southern California, 1845 N. Soto Street, Los Angeles, CA 90032 USA

**Keywords:** Pornography, Depression, Anxiety, Epidemiology, Gender differences

## Abstract

Young adulthood is a critical development period when mental health problems such as anxiety and depression become more prevalent. Likewise, there is evidence to suggest that depression and anxiety may lead to increased pornography usage. We examined the association of depression and anxiety symptoms with pornography viewing frequency among a prospective cohort of young adults (*n* = 1864) from California. Multinomial logistic regression models estimated the association of depression symptoms only, anxiety symptoms only, and comorbid depression and anxiety with pornography viewing frequency (never, < 3 times/month, one to several times/week, one to several times/day) at a 6-month follow-up. Models adjusted for gender, sexual orientation, sexual satisfaction, and adverse childhood experiences. Participants with comorbid depression and anxiety (vs. no depression or anxiety symptoms) had 2.72 (95% CI: 1.66–4.46) times the odds of frequent pornography viewing (one to several times/day) compared to never watching pornography. There was an association of depression symptoms only with frequent pornography viewing but it did not reach statistical significance (OR: 1.95, 95% CI: 0.78–4.89). Anxiety symptoms alone (vs. no depression and anxiety symptoms) were not associated with pornography viewing at any frequency in the full sample. However, in gender-stratified models, anxiety symptoms alone were associated with pornography viewing among women (OR: 1.44. 95% CI: 1.00–2.07), but not men (1.12, 95% CI: 0.65–1.96). Findings suggest comorbid depression and anxiety symptoms are associated with frequent pornography viewing among young adults, and anxiety symptoms alone are associated with pornography viewing among women, but not men.

## Introduction

Young adulthood is a critical stage in physical and mental development when many individuals experience the onset of mental health issues, such as anxiety and depression (Jurewicz, 2015). Mental health issues like depression and anxiety are often precursors to the development of various behavioral and emotional problems, including addictive behaviors such as excessive pornography viewing (Paus et al., 2008; Sun & Chang, 2021). Consistent with mood management theory (Zillmann, 1988), pornography might provide a temporary escape from emotional distress, leading young adults with symptoms of anxiety or depression to watch pornography more frequently than those without anxiety or depression (Cardoso et al., 2022; Ybarra & Mitchell, 2005).

While many young adults view pornography in a recreational, non-problematic manner, some develop problematic pornography use characterized by compulsive or addictive patterns of use that can negatively impact other areas of life (Duffy et al., 2016; Štulhofer et al., 2010). Though frequent pornography use does not always constitute problematic use, greater exposure and more frequent viewing of pornography may increase the risk of transitioning to problematic pornography use by enhancing cue reactivity, cravings, and diminished self-control around pornography (Bőthe et al., 2021, 2022; Wéry & Billieux, 2016; Wordecha et al., 2018). Understanding the role of depression and anxiety as antecedents of frequent pornography usage can help inform interventions that seek to enhance pornography media literacy and reduce potential adverse outcomes related to pornography use among young adults (Malamuth & Huppin, 2005).

Prior cross-sectional studies have identified psychological factors associated with frequent pornography use among adolescents and young adults, including depressive symptoms, negative emotional states, and poor emotional self-regulation abilities (Paul & Shim, 2008; Reid et al., 2011; Willoughby et al., 2014, 2018). In longitudinal studies, findings are mixed regarding mental health symptoms as antecedents of frequent pornography viewing and/or problematic pornography use. For example, in a study of Dutch adolescents, Doornwaard et al. (2017) found depressive symptoms predicted subsequent compulsive pornography use at a 6-month follow-up. Rousseau et al. (2021) investigated theoretical antecedents of problematic pornography use among male adolescents in Croatia, reporting that baseline higher levels of negative emotions and impulsivity predicted higher levels of problematic pornography use 3 years later. Conversely, Kohut and Štulhofer (2018) and Štulhofer et al. (2019) both found no significant prospective link between depression and anxiety symptoms and pornography viewing frequency among Croatian adolescents. Additionally, Bőthe et al. (2022) found that pornography use frequency and problematic pornography use among Hungarian adolescents remained stable before and after the COVID-19 pandemic, despite increases in stress and mental health issues during this time. Longitudinal evidence on the association of anxiety symptoms specifically (in the absence of depression) with pornography use outcomes remains limited.

Importantly, little research has attempted to distinguish whether certain mental health symptoms (e.g., depressive vs. anxious) have stronger correlations with frequent pornography viewing. While depression and anxiety are related and often co-occurring, these two conditions have distinct symptoms and patterns that may differentially influence pornography viewing behaviors. Depression is characterized by persistent feelings of sadness, anhedonia, low self-worth, and diminished motivation (American Psychiatric Association, 2013). Individuals with depression often use avoidant coping strategies and may turn to pornography viewing as a form of escape and pleasure-seeking to alleviate negative emotions (Cardoso et al., 2022; Ybarra & Mitchell, 2005). The continuous reinforcement and novelty provided by pornography could become a maladaptive coping mechanism for those with depression. In contrast, anxiety involves excessive worry, physiological hyperarousal, and feelings of restlessness (Hoge et al., 2012). Pornography may be used as a mechanism to relieve anxious arousal through sexual stimulation (Muris et al., 2008). Anxiety is also linked to impulsivity, which could increase risky sexual behaviors like problematic pornography viewing (Antons & Brand, 2018; Bőthe et al., 2020; Egan & Parmar, 2013). Although anxiety symptoms could plausibly lead to increased pornography use, studies on mental health determinants of pornography viewing have primarily focused on depression or combined depression and anxiety symptoms together. Evidence on whether anxiety symptoms are equally, more, or less strongly associated with pornography viewing relative to depression symptoms among young adults is limited (Ma, 2019; Mattebo et al., 2018).

Additionally, many prior studies on mental health and pornography use were conducted among predominately White and/or European samples (Bőthe et al., 2022; Doornwaard et al., 2017; Kohut & Štulhofer, 2018), which limits generalizability of findings. This is a concern given that both mental health and sexual behavior varies across racial/ethnic and cultural subgroups (Perry & Schleifer, 2017). Furthermore, few prior longitudinal studies examine mental health antecedents of pornography use in young adulthood—a critical period of development during which pornography use and mental health symptoms increase. The current study aims to enhance the existing literature by examining the longitudinal association of mental health symptoms with subsequent pornography viewing among a demographically-diverse sample of young adults, with a focus on differentiating associations of depression versus anxiety symptoms.

We investigated the relationship between mental health symptoms and subsequent pornography viewing using longitudinal data from a prospective cohort of young adults from Southern California. We aimed to determine whether symptoms of depression, anxiety, or comorbid depression and anxiety were associated with the frequency of pornography watching. We also examined effect modification by gender, as prior research suggests that males tend to watch pornography more frequently than females (Griffiths, 2012; Sun et al., 2016; Willoughby et al., 2014) and the association between mental health symptoms and pornography may differ by gender (Bőthe et al., 2021; Kohut & Štulhofer, 2018; Willoughby et al., 2014; Yu et al., 2022). We hypothesized that young adults who showed symptoms of depression and/or anxiety would have a higher risk of more frequent pornography watching compared to those who do not show symptoms of depression and/or anxiety. We did not have a priori hypotheses on whether depression or anxiety symptoms would confer greater risk or hypotheses on the specific magnitude or direction of gender differences.

## Method

### Participants

Data were from the Happiness & Health (H & H) Study, a prospective cohort study of social and emotional health, health behaviors, and substance use. Ninth graders from 10 Los Angeles area high schools selected for sociodemographic diversity were enrolled in H & H in 2013 (Leventhal et al., 2015). Waves 1–8 were collected semi-annually each semester during high school. Participants then reconsented as adults (after high school) and completed online surveys approximately every 6–12 months during young adulthood. Wave 10 (collected March-September 2020) is the baseline for the current analysis and Wave 11 (collected January-June 2021) was used to assess prospective follow-up data.

**Fig. 1:**
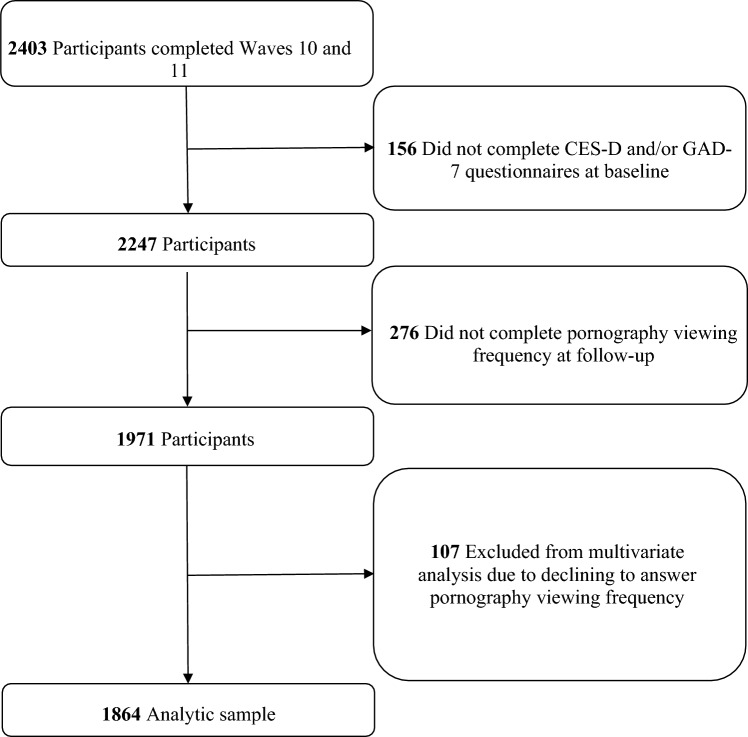
Flowchart of inclusion and exclusion criteria

The analytic sample included participants with non-missing data on mental health symptoms at baseline (Wave 10) and pornography viewing at follow-up (Wave 11). Pornography viewing was only assessed on the Wave 11 survey and was not measured at the baseline (Wave 10) survey. Among 2403 participants who completed surveys at both baseline and follow-up, 2247 (93.5%) completed the questions on mental health symptoms at baseline. Of those participants, 1971 answered pornography questions at follow-up. The final analytic sample was 1864 after further excluding 107 participants (5.4%) who responded “prefer not to answer” in response to pornography viewing frequency (Fig. 1).

### Measures

#### Exposure: Depression and Anxiety Symptoms

Depression and anxiety symptoms were assessed at baseline (Wave 10) using the 10-item Center for Epidemiological Studies Depression Scale-Short Form (CESD-10) and 7-item General Anxiety Disorder-7 Form (GAD-7), respectively. We used validated clinical thresholds to define the presence of depressive or anxiety symptoms (Spitzer et al., 2006; Zhang et al., 2012). A composite score of 10 or greater on the CESD-10 indicated the presence of significant depressive symptoms and a composite score of 10 or greater on the GAD-7 indicated the presence of significant anxiety symptoms (Spitzer et al., 2006; Zhang et al., 2012). Our primary exposure variable was a combined mutually exclusive four-level symptom exposure variable: (1) neither depressive nor anxiety symptoms (reference); (2) depressive symptoms only; (3) anxiety symptoms only, and (4) both depressive and anxiety symptoms. In secondary analyses, we examined depression and anxiety symptoms as separate continuous score variables.

#### Outcome: Pornography Viewing

Frequency of pornography viewing was assessed at follow-up (Wave 11, the first and only wave in which the measure was included on surveys), approximately 6 months after baseline. Participants were asked to, “indicate how often you watch videos of people engaged in sexual activity on the internet or from other sources” (responses: never, less than once per month, 1–3 times per month, once per week, several times per week, once every day, two or more times per day, prefer not to answer). We excluded participants who did not report their pornography-viewing frequency at follow-up or who reported “prefer not to answer.” Due to sparse data (i.e., *n* < 5) in some response options, we combined the remaining categories into a four-level pornography frequency variable for primary analyses (never, < 3 times/month, one to several times/week, one to several times/day). A binary pornography viewing variable was also created, with the levels: never watch pornography, and ever watch pornography at any frequency.

#### Covariates

We identified possible confounders as factors associated with both mental health symptoms and pornography viewing in prior literature, including gender, sexual identity, sexual satisfaction, and adverse childhood experiences (ACEs) (Svedin et al., 2023).

Both gender and sexual identity have associations with pornography viewing frequency and mental health symptomology. Males and sexual and gender minority individuals generally report greater pornography frequency (Bőthe et al., 2019; Griffiths, 2012; Sun et al., 2016), and females and sexual and gender minority individuals report worse mental health symptoms (Herek & Garnets, 2007; Riecher-Rössler, 2017). Gender was measured at baseline using a composite four-level variable with the following categories: man, woman, transgender or non-binary, and prefer not to answer. The transgender or non-binary category included participants who identified as a transgender male, transgender female, gender variant/non-binary, or another gender identity not listed. Sexual identity was measured at baseline using a composite 3-level variable with the following categories: heterosexual, sexual minority, or prefer not to answer. Sexual minority identity included participants who identified as asexual, bisexual, gay, lesbian, pansexual, queer, questioning, or another sexual identity not listed.

Low sexual satisfaction can lead to poor mental health outcomes (Carcedo et al., 2020), and is also associated with pornography frequency in prior literature (Wright et al., 2019). Sexual satisfaction at follow-up was measured with three separate questions using a Likert scale (agree, neither agree nor disagree, disagree, prefer not to answer) in response to the statements “I feel satisfied with my sex life”, “I feel distressed or worried about my sex life” and “I avoided sex because of sexual difficulties of self/partner.” All three sexual satisfaction variables were highly correlated (*r*s = 0.53–0.75, *p* < 0.0001), and thus a composite variable was created that assigns a numerical value to each answer and takes the average across all three variables.

ACEs like abuse, neglect, and household dysfunction may contribute to increased pornography viewing behaviors in young adulthood (Noll, 2021). Childhood trauma is linked to difficulties with emotional regulation and higher rates of risky sexual behaviors, which could manifest as using pornography in unhealthy ways to cope with negative emotions or memories from ACEs (Handley et al., 2020). ACEs are also well known to cause adverse mental health issues during young adulthood (Hughes et al., 2017; Petruccelli et al., 2019). ACEs were assessed at follow-up using the Adverse Childhood Experiences Questionnaire (ACE-Q) in which participants recalled adverse events experienced prior to age 18 (Felitti et al., 1998). Participants who answered yes to the question: “Did an adult or person at least 5 years older than you ever touch or fondle you or have you touched their body in a sexual way? Or, try to or actually have oral, anal, or vaginal sex with you?” were classified as having experienced a sexual ACE regardless of whether they answered yes to any of the other ACE questions. Participants who answered yes to at least one of the non-sexually related questions were classified as having experienced a non-sexual ACE. Participants who answered no to every question were classified as not having experienced an ACE, sexual or non-sexual.

In addition to the aforementioned confounders, we included a race/ethnicity variable (Hispanic/Latinx, Non-Hispanic/Latinx, American Indian/ Alaska Native, Asian, Black/African American, Native Hawaiian/ Pacific Islander, White, Multi-racial/Multi-ethnic, Another race/unknown) to describe the sample. For variables with a “prefer not to answer” response option (gender, sexual identity, sexual satisfaction), participants with missing data on covariates were combined with participants who selected “prefer not to answer” into a single “missing/prefer not to answer” category. Other covariates such as ACEs and race/ethnicity did not have “prefer not to answer categories” and were not missing any data after other exclusions.

### Statistical Analysis

Descriptive statistics of the exposure, outcomes, and covariates were examined. Multivariable multinomial logistic regression models were fitted to estimate the crude and adjusted odds ratios for associations of symptoms of depression and anxiety at baseline with frequency of pornography viewing at follow-up, adjusting for gender, sexual identity, sexual satisfaction, and ACEs. The outcome reference in the multinomial model was never watched pornography.

In secondary analyses, we examined the association of continuous depression and anxiety scores with pornography frequency simultaneously in the same model. We also assessed for potential effect modification of the association of depression and anxiety with the frequency of pornography viewing by gender. We chose to assess for effect modification by gender because there is often greater pornography use among males and known gender differences in mental health prevalence (Maheux et al., 2021; Mattebo et al., 2018). To assess for effect modification by gender, we tested a formal interaction term between mental health symptoms x gender and subsequently stratified models by male versus female; participants who reported a transgender/non-binary gender or preferred not to report their gender were excluded due to sparse data. For stratified models, we used the binary outcome of ever versus never pornography viewing in binary logistic regression models because the pornography viewing strata became sparse after stratifying for gender (i.e., 0 cases in some strata).

## Results

### Descriptive Analyses

The demographic characteristics of the study population stratified by mental health symptoms are presented in Table 1. The sample was predominantly Hispanic/Latinx (46.4%) and Asian (19.1%), with a mean age of 21.2 years at baseline. Overall, 43.1% of the sample reported both anxiety and depressive symptoms, 5.2% only depressive symptoms, 19.7% only anxiety symptoms, and 32.0% no depressive or anxiety symptoms. Compared to participants with no depression or anxiety symptoms, those with both anxiety and depression were more likely to be women (65.6% vs. 47.3%), identify as a sexual minority identity (31.0% vs 11.1%), and report sexual (13.8% vs. 5.7%) and non-sexual ACEs (57.4% vs. 40.9%).

**Table 1:** Characteristics of the young adult sample (*N* = 1864)

	Full sample	No depression or anxiety symptoms^a^	Only depression symptoms^a^	Only anxiety symptoms^a^	Depression and anxiety symptoms^a^
Total N	1864	596	98	367	803
*Gender*					
Man	695 (37.2)	302 (50.7)	45 (45.9)	113 (30.8)	235 (29.3)
Woman	1108 (59.4)	282 (47.3)	51 (52.0)	248 (67.6)	527 (65.6)
Transgender or non-binary^b^	37 (2.0)	5 (0.8)	1 (1.0)	4 (1.1)	27 (3.4)
Missing/Prefer not to answer	24 (1.3)	7 (1.2)	1 (1.0)	2 (0.5)	14 (1.7)
*Race/Ethnicity*					
Hispanic/Latinx	865 (46.4)	282 (47.3)	51 (52.0)	172 (46.9)	360 (44.8)
American Indian/Alaska native	10 (0.01)	6 (1.0)	0 (0.0)	2(0.5)	2 (0.3)
Asian	354 (19.0)	114 (19.1)	19 (19.4)	61 (16.6)	161 (20.1)
Black or African American	78 (4.2)	27 (4.5)	6 (6.1)	9 (2.5)	36 (4.5)
Native Hawaiian/Pacific Islander	72 (3.9)	23 (3.9)	6 (6.1)	11 (3.0)	32 (4.0)
White	309 (16.6)	85 (14.3)	11 (11.2)	73 (19.9)	140 (17.4)
Multi-racial/Multi-ethnic	118 (6.3)	36 (6.0)	3 (3.1)	29 (7.9)	50 (6.2)
Other/unknown	57 (3.1)	23 (3.9)	2 (2.0)	10 (2.7)	22 (2.7)
*Sexual identity*					
Heterosexual	1434 (76.9)	517 (86.7)	78 (79.6)	303 (82.6)	536 (66.8)
Sexual minority^c^	397 (21.3)	66 (11.1)	20 (20.4)	62 (16.9)	249 (31.0)
Missing/Prefer not to answer	33 (1.8)	13 (2.2)	0 (0.0)	2 (0.5)	18 (2.2)
Sexual satisfaction composite score^d^	1.80 (1.24)	1.86 (1.30)	1.82 (1.24)	1.90 (1.22)	1.71 (1.19)
*Adverse childhood experiences (ACE)*					
Had sexual ACE	195 (10.5)	34 (5.7)	9 (9.2)	35 (9.5)	116 (13.8)
Had non-sexual ACE	1013 (54.3)	244 (40.9)	49 (50.0)	214 (58.3)	461 (57.4)
Did not have ACE	756 (40.6)	318 (53.4)	40 (40.8)	118 (32.2)	231 (28.8)
*Pornography viewing frequency*^e^					
Never	632 (33.9)	237 (39.8)	31 (31.6)	141 (38.4)	223 (27.8)
Less than once per month	297 (15.9)	86 (14.4)	21 (21.4)	62 (16.9)	128 (15.9)
One to three times per month	287 (15.4)	82 (13.8)	14 (14.3)	54 (14.7)	137 (17.1)
Once a week	158 (8.5)	53 (8.9)	4 (4.1)	28 (7.6)	73 (9.1)
Greater than once a week	322 (17.3)	83 (13.9)	17 (17.4)	64 (17.4)	158 (19.7)
Once per day	120 (6.4)	43 (7.2)	8 (8.2)	14 (3.8)	55 (6.9)
Greater than once per day	48 (2.6)	12 (2.0)	3 (3.1)	4 (1.1)	29 (3.6)

Estimates presented as column *n* (%) unless otherwise noted

^a^Participants were considered to exhibit depression symptoms with a CESD score ≥ 10, and anxiety symptoms with a GAD score ≥ 10

^b^Participants who identified as transgender male, transgender female, gender variant/non-binary, or another gender

^c^Participants who identified as asexual, bisexual, gay, lesbian, pansexual, queer, questioning, or another sexual identity

^d^Estimates presented as column mean (SD). Lower scores correlate to lower sexual satisfaction. The minimum possible score is 0 and the maximum possible score is 3.

^e^Collapsed for primary analyses: never, < 3 times/month, one to several times/week, one to several times/day

Overall, 33.9% of participants reported never watching pornography at follow-up, 31.3% reported watching pornography 3 times or less per month, 25.8% once to several times per week, and 9.0% once to several times/day (Table 1). In descriptive analyses, participants who reported comorbid depression and anxiety were more likely to report watching pornography at most frequencies compared to participants with no symptoms.

### Primary Analyses

Table 2 displays the results of multinomial logistic regression models assessing pornography viewing frequency as a function of anxiety and depression symptoms. In adjusted models, the presence of both depression and anxiety symptoms (vs. no depression and anxiety symptoms) was associated with greater odds of watching pornography at all frequencies versus never watching (3 times or less per month: adjusted odds ratio[aOR] = 1.61, 95% CI: 1.20–2.15; Once to several times per week: aOR= 2.34, 95% CI: 1.71, 3.35; Once to several times per day: aOR = 2.72, 95% CI: 1.66–4.46). Adjusted odds ratios for participants with only depressive symptoms (versus no symptoms) also showed an increase in the odds of viewing pornography once to several times per day (aOR = 1.95, 95% CI: 0.78–4.89), and three times or less per month (aOR = 1.57, 95% CI: 0.91–2.69) but confidence intervals were wide and included the null. There was no association of depression symptoms alone with pornography viewing once to several times per week. Exclusive anxiety symptoms were associated with slightly elevated (but not significant) odds of pornography viewing several times per week (aOR = 1.43, 95% CI: 0.98–2.10). However, there was little evidence for an association of exclusive anxiety symptoms with frequent (i.e., once to several times per day) or infrequent (i.e., three times or less per month) pornography viewing.

**Table 2:** Association of symptoms of anxiety and depression with frequency of pornography viewing (*n* = 1864)

	Frequency of pornography viewing at follow-up^a^					
	3 times or less per month		Once to several times per week		Once to several times per day	
	Crude OR (95%CI)	aOR^b^ (95%CI)	Crude OR (95%CI)	aOR^b^ (95%CI)	Crude OR (95%CI)	aOR^b^ (95%CI)
*Categorical baseline symptoms*						
No symptoms of depression and anxiety	Ref	Ref	Ref	Ref	Ref	Ref
Only depression symptoms	1.59 (0.95–2.69)	1.57 (0.91–2.69)	1.18 (0.65–2.14)	1.20 (0.61–2.37)	1.53 (0.72–3.23)	1.95 (0.78–4.89)
Only anxiety symptoms	1.16 (0.85–1.59)	1.20 (0.80–1.57)	1.14 (0.81–1.59)	1.43 (0.98–2.1)	0.55 (0.31–0.97)	0.74 (0.38–1.43)
Depression and anxiety symptoms	1.67 (1.29–2.19)	1.61 (1.20–2.15)	1.81 (1.37–2.39)	2.34 (1.71–3.35)	1.62 (1.10–2.39)	2.72 (1.66–4.46)
*Continuous baseline symptoms*^c^						
Depression symptoms	1.04 (1.01–1.08)	1.05 (1.02–1.08)	1.05 (1.02–1.08)	1.06 (1.03–1.10)	1.10 (1.06–1.15)	1.12 (1.07–1.18)
Anxiety symptoms	0.99 (0.96–1.02)	0.98 (0.95–1.01)	0.99 (0.95–1.02)	1.00 (0.96–1.04)	0.94 (0.90–0.99)	0.97 (0.91–1.02)

^a^Outcome reference in multinomial logistic regression model is “never watching pornography”

^b^Adjusted for gender, sexual orientation, sexual satisfaction, and ACEs

^c^Models included depression and anxiety symptom scores as separate continuous variables in the same model

### Secondary Analyses

In the model examining continuous exposure variables for depression and anxiety symptom scores simultaneously, a greater depression symptom score was positively associated with viewing pornography at all frequencies versus never watching (3 times or less per month: aOR = 1.05, 95% CI: 1.02–1.08; Once to several times per week: aOR = 1.06, 95% CI: 1.03–1.10; Once to several times per day: aOR = 1.12, 95% CI: 1.07–1.18 (Table 2). However, anxiety symptom scores were not associated with pornography viewing at any frequency.

In moderation analyses, the *p* value for the interaction term between mental health symptoms x gender for the outcome of any pornography viewing was not statistically significant (*p* = 0.9719) (Table 3). Stratifying by gender revealed that men with comorbid depression and anxiety symptoms had 2.72 (95% CI: 1.60–4.62) times the odds of any pornography watching compared to men without depression and anxiety symptoms. Men with only depression or only anxiety were not more likely to watch pornography compared to men with no anxiety and depression symptoms. Similarly to men, women with only depression symptoms were not significantly more likely to watch pornography compared to women with no depression or anxiety symptoms. However, women with comorbid depression and anxiety (aOR = 1.94, 95% CI: 1.42–2.61) and women with only anxiety (aOR = 1.44, 95% CI: 1.00–2.07) were more likely to watch pornography compared to women with no symptoms.

**Table 3:** Association of mental health symptoms with pornography viewing stratified by gender (*n* = 1803)^a^

	Men	Women
Baseline mental health symptoms	aOR^a^ (95%CI)	aOR^a^ (95%CI)
No symptoms of depression and anxiety	Ref	Ref
Only depression symptoms	1.29 (0.56–2.70)	1.70 (0.91–3.16)
Only anxiety symptoms	1.12 (0.65–1.96)	1.44 (1.00–2.07)
Depression and anxiety symptoms	2.72 (1.60–4.62)	1.94 (1.42–2.66)

Interaction *p* value = 0.9719

^a^Excluded transgender/non-binary participants and those who preferred not to report their gender due to sparse cells

^b^Pornography viewing at any frequency compared to never viewing pornography

^b^Adjusted for ACEs, sexual orientation, and sexual satisfaction

## Discussion

Our study found that among a diverse sample of young adults from Southern California, those with comorbid symptoms of both depression and anxiety and those with depression symptoms alone were more likely to report frequent pornography viewing (once or more per day) compared to those without depression or anxiety symptoms. Higher depression symptom scores were also positively associated with pornography viewing frequency, after adjusting for comorbid anxiety symptoms. Conversely, there was little evidence of anxiety symptoms alone being associated with frequent pornography viewing in the overall sample. However, we observed differences in associations when considering gender; anxiety symptoms (without the presence of depression) were associated with any pornography viewing for women, but not men.

Our findings add to the body of literature demonstrating an association between mental health symptoms and increased pornography consumption (Grubbs & Kraus, 2021; Rousseau et al., 2021). Specifically, our study revealed an association between depression symptoms and pornography consumption, identifying a potentially vulnerable population (young adults with depression symptoms) that has a higher risk of being exposed to pornographic content. Other longitudinal studies have similarly found an association between depressive symptoms and pornography use in adolescent populations. For example, a longitudinal study by Rousseau et al. (2021) showed that higher levels of negative emotions at baseline predicted higher levels of subsequent problematic pornography usage among Croation boys, and Doornwaard et al. (2017) found that depression predicted higher pornography usage among Dutch adolescents aged 16–19. We provide additional compelling evidence that mental health symptoms are linked with pornography viewing among a cohort of young adults from California, with possible differences in associations observed across gender and type of emotional disorder.

In the current study, increased depression symptoms, but not anxiety symptoms, were associated with greater pornography use frequency. The difference in the likelihood of frequently viewing pornography between participants with symptoms of depression versus those with symptoms of anxiety could be driven by the way that depression and anxiety symptoms manifest among young adults. Depression as a disorder is often characterized by both anhedonia (e.g., reduced feelings of pleasure) and negative emotions (e.g., sadness) (Fitzgerald, 2013; Fresán & Berlanga, 2013). Because depression is often accompanied by anhedonia, individuals with depression disorders may be more likely to seek out stimulating behaviors that cause energy, euphoria, and excitement, such as pornography viewing (Levin et al., 2019). In contrast, anxiety is generally characterized by negative emotions, physiological hyperarousal, and feelings of restlessness, but not necessarily anhedonia (Weiner, 2007). Therefore, those with anxiety may seek out calming behaviors that quell anxious thoughts but may not be more likely than those without mental health symptoms to seek out stimulating or pleasure-inducing behaviors such as pornography (Labroo & Rucker, 2010). Our findings are consistent with at least one prior cross-sectional study among adults that similarly found depression symptoms, but not anxiety symptoms, to be predictors of problematic pornography use (Borgogna et al., 2018).

However, in the current study our moderation analyses did show anxiety symptoms alone were associated with pornography viewing at any frequency among women (but not men). Pornography viewing may serve as a maladaptive coping mechanism for women to relieve anxious symptoms such as psychological arousal through distraction (Bőthe et al., 2019; Laier & Brand, 2017; Muris et al., 2008). It is not entirely clear why the association was only observed among women in this sample, though this finding may be driven lower overall pornography use frequency among women; relative associations (e.g., odds ratios) are often stronger among subsamples with a lower risk of the outcome (Rothman & Poole, 1988). Additionally, the interaction term was not significant and thus gender differences may simply be due to random error. Additional research is needed to further confirm and explore potential mechanisms underlying differences in the association of mental health symptoms with pornography viewing by symptom type and gender.

Our study has several strengths, including distinguishing associations of depression versus anxiety symptoms with pornography use in a sample of primarily Hispanic/Latinx and Asian young adults. Additionally, the prospective design allowed us to ensure assessment of mental health symptoms occurred prior to the assessment of pornography frequency. However, the study has several limitations. We cannot preclude the possibility of reverse causation (i.e. that pornography use affected mental health symptoms) due to the lack of a baseline measure of pornography viewing. We also did not measure problematic pornography use or the type of pornography viewed by participants. Additionally, pornography assessment only focused on video (and not on images); this may have resulted in an underestimate of the prevalence of pornography viewing. Future research could assess associations with different types or genres of pornography to gain a better understanding of the link between mental health and various types of pornography. The study also relied on self-reported data, which may have caused non-differential misclassification of certain variables, particularly sensitive questions about sexual satisfaction and pornography viewing, which would likely bias our effect estimates toward the null. Additionally, adjusting for sexual satisfaction at the follow-up wave might result in over-adjustment bias (i.e., conditioning on a mediator) because sexual satisfaction at follow-up could potentially be affected by mental health status at baseline. Due to sparse data, our moderation analyses used a binary outcome of any versus no pornography viewing, rather than the outcome of frequent pornography viewing. Finally, ~18% of participants were excluded due to missing data, which could bias results if those excluded differed from included participants on mental health and pornography viewing.

Importantly, this study identifies mental health as a possible “risk factor” for pornography viewing, however, we acknowledge that more research is needed on positive traits that may predict pornography viewing. The exclusion of positive characteristics as predictors of pornography use in the literature may inadvertently reinforce negative stereotypes and stigmatize individuals who use pornography. Such stigma can present significant obstacles to access to support services and perpetuate feelings of shame and guilt often associated with pornography use. Adopting a comprehensive approach that examines both risk and protective factors as predictors is crucial to promote a more nuanced understanding of pornography use.

This study reveals an association between mental health symptoms and frequent pornography viewing in young adults. Mental health problems have increased among young adults over the past decade and are currently at an all-time high (Goodwin et al., 2022). Results suggest that individuals with higher levels of depressive symptoms and comorbid depressive and anxious symptoms are more likely to consume pornographic content more frequently. Findings reinforce that pornography viewing may serve as a maladaptive coping mechanism for depressive symptoms by providing temporary relief from emotional distress through stimulation and novelty-seeking. It is imperative for educators, parents, and mental health professionals to be aware of the increased likelihood of pornography consumption by depressed young adults. Educational programs could be designed to inform young people about the potentially harmful effects of problematic pornography use, while mental health professionals could offer counseling on pornography media literacy skills and managing depression and negative emotions with healthy coping mechanisms.

## Data Availability

This paper uses data from the USC Health & Happiness Study. The data that support the findings of this study are available from the corresponding author upon reasonable request.

## Code Availability

Analyses were conducted in SAS v9.4. Code is available upon reasonable request.
